# Waterpipe smoking and cardiovascular mortality: Challenging the safety Myth

**DOI:** 10.1016/j.ajpc.2026.101559

**Published:** 2026-03-18

**Authors:** Rana Jaber

**Affiliations:** Public Health, School of Health Studies, College of Health and Human Sciences, Northern Illinois University, DeKalb, IL 60115, USA

Editorial

Cardiovascular disease (CVD) remains the leading cause of death worldwide, with tobacco use among the most important modifiable risk factors. While tobacco control efforts have successfully reduced cigarette smoking rates, new forms of tobacco use such as waterpipe and e-cigarettes have emerged and gained popularity, particularly among youth and young adults. Waterpipe smoking, also known as hookah, shisha, narghile, argileh, or hubbly bubbly, is a traditional form of tobacco use in the Middle East and parts of South Asia, where it is embedded in social and cultural practices. Its global popularity has increased dramatically over the past two decades, driven by appealing flavors, social acceptability, and the persistent but erroneous perception that waterpipe smoking is less harmful than cigarettes because smoke passes through water believed as filtered before inhalation [1].

The recent prospective cohort study “Waterpipe Smoking and Risk of Cardiovascular Disease Mortality” offers critical new evidence linking waterpipe use with increased risk of CVD mortality, directly challenging the misperception of reduced harm. Using data from the ongoing Hanoi Prospective Cohort Study (HPCS), which enrolled over 52,000 participants from nine communities in Northern Vietnam between 2007 and 2008, investigators included 35,646 Vietnamese adults aged 18 years and older to examine the association between ever-smoking waterpipe and fatal CVD outcomes. Compared to individuals who never smoked waterpipe, those who ever smoked waterpipe had a **57% higher risk of CVD mortality** (HR, 1.57; 95% CI, 1.16–2.13) and a **44% higher risk of cerebrovascular mortality** (HR, 1.44; 95% CI, 1.08–1.93). Individuals reporting dual waterpipe and cigarette smoking had a **64% greater CVD mortality risk** versus never smokers (HR, 1.64; 95% CI, 1.14–2.36). Notably, among waterpipe smokers who also reported ever drinking alcohol and heavy long-term cigarette smoking, the risk of CVD death was more than double that of waterpipe smokers without these exposures (HR, 2.05; 95% CI, 1.36–3.08). These findings underscore the potentially additive and synergistic cardiovascular toxicity of multiple substance exposures and reinforce the importance of comprehensive prevention strategies [2]

The reduced-harm perception of waterpipe stems partly from its unique smoking mechanism of heating flavored tobacco with charcoal to produce smoke that passes through water before inhalation. However, toxicological studies clearly indicated that this process does **not** effectively filter harmful constituents. Waterpipe smoke contains high concentrations of nicotine, carbon monoxide, particulate matter, heavy metals, and carcinogenic organic compounds, many at levels like or exceeding those found in cigarette smoke [3]. The charcoal used to heat the tobacco adds significant quantities of carbon monoxide and polycyclic aromatic hydrocarbons both strong contributors to cardiovascular toxicity [1].

The unique pattern of waterpipe smoking contributes to the increased likelihood of exposure to its smoke toxic substances. Unlike cigarette smoking that lasts 5 min, waterpipe smoking sessions may last 60 min during which users inhale larger volumes of smoke [1]. Previous experimental studies indicate that a single 45 min waterpipe smoking session may expose users up to 36.5 times more nicotine-free dry particulate matter (TAR) than a single cigarette smoking [4]. Therefore, the repeated inhalation of those toxicants may predict long-term adverse health outcomes as those shown by this longitudinal study [2]

Understanding the biological mechanisms linking waterpipe smoking with CVD mortality strengthens the plausibility of the observed associations. Waterpipe smoke induces nicotine-mediated sympathetic activation, increases heart rate and blood pressure acutely, and promotes carbon monoxide–mediated impairment of oxygen delivery to tissues. Fine particulate matter and toxic organic compounds trigger oxidative stress, endothelial dysfunction, and vascular inflammation that are considered underlying processes in atherogenesis. Experimental studies demonstrate that acute waterpipe smoking increases heart rate and blood pressure while impairing endothelial function, indicating immediate adverse vascular effects [5]. Additionally, waterpipe smokers demonstrate adverse cardiometabolic profiles, including higher triglycerides, low-density lipoprotein cholesterol, fasting glucose, resting heart rate, and lower high-density lipoprotein cholesterol compared with non-smokers, and each is a well-established risk factor for CVD. Observational studies have also linked waterpipe use to coronary artery disease and myocardial infarction, aligning with the long-term mortality risk identified in recent prospective research [6,7]

An important observation from the HPCS analysis is the elevated CVD mortality among individuals reporting dual use of waterpipe and cigarettes relative to exclusive use of either product. Dual use likely represents greater cumulative exposure to nicotine and toxicants, resulting in additive or synergistic cardiovascular toxicity. The higher CVD mortality observed among individuals reported ever smoked waterpipe, smoked at least 20 cigarettes daily, and ever drank alcohol, should be carefully interpreted. Alcohol was measured as binary “ever use,” which does not capture drinking intensity and may mask substantial variation in consumption within this subgroup. This is particularly relevant given the ongoing debate regarding the cardiovascular protective effects of light-to-moderate alcohol use. Beyond these physiological considerations, the co-occurrence of these behaviors suggests clustering of cardiovascular risk behavior [8].

These findings carry significant clinical and public health implications. Clinicians should routinely screen for waterpipe use as part of comprehensive tobacco exposure assessment. Early identification of waterpipe users creates opportunities for targeted cessation counseling and risk reduction. Regulatory frameworks should expand beyond cigarettes to encompass waterpipe tobacco products, which often escape restrictions such as flavor bans, standardized labeling, health warnings, and taxation. Importantly, educational initiatives targeting adolescents, young adults, and communities where waterpipe use is prevalent are urgently needed to correct misperceptions about waterpipe safety and raise awareness of its substantial toxicant exposure and cardiovascular harms.

The longitudinal design of the HPCS and its large sample size represent key strengths, providing temporal evidence linking waterpipe smoking to cardiovascular mortality. However, future studies should examine the dose–response relationships by incorporating detailed measures of frequency, duration, and intensity of waterpipe exposure, including patterns for emerging products such as electronic waterpipe. Investigations assessing differences in susceptibility by sex, age, and geographic populations are critical as waterpipe use expands globally. Additionally, more comprehensive assessments of alcohol consumption and other co-occurring behaviors will be essential to disentangle the joint effects of waterpipe smoking, any substance use, and other lifestyle risk factors on cardiovascular outcomes.

In conclusion, the accumulating evidence indicates that waterpipe smoking is a significant and underrecognized threat to cardiovascular health. The prospective findings reported by Le and colleagues provide compelling evidence that waterpipe use is associated with increased cardiovascular mortality, particularly among individuals who also smoke cigarettes. These results directly challenge the persistent misconception that waterpipe smoking is a safer alternative to cigarettes and highlight the urgent need for stronger clinical, regulatory, and public health responses to reduce the burden of tobacco-related cardiovascular disease.

## Declaration of generative AI and AI-assisted technologies

After completing the initial draft of this work, the author used ChatGPT to help maintain the word count within the limits. After using this tool, the author reviewed and edited the content as needed and therefore takes full responsibility for the content of the published article.

## Statement of authorship

RJ conceptualized the commentary, conducted the literature review, and wrote and approved the final manuscript.

## Author’s agreement

The author has seen and approved the final version of the manuscript being submitted. They warrant that the article is the authors' original work, hasn't received prior publication and isn't under consideration for publication elsewhere.

## CRediT authorship contribution statement

**Rana Jaber:** Writing – review & editing, Writing – original draft, Conceptualization.

## Declaration of competing interest

The authors declare that they have no known competing financial interests or personal relationships that could have appeared to influence the work reported in this paper.
